# Development of peribronchiolar fibrosis is associated with local immune and MAPK pathway activation in a murine model of deployment-related constrictive bronchiolitis

**DOI:** 10.3389/fpubh.2026.1761783

**Published:** 2026-03-16

**Authors:** Seagal Teitz-Tennenbaum, Kayla N. Marinetti, Anutosh Ganguly, Brian Song, Helen Hicks, John J. Osterholzer

**Affiliations:** 1 Research Service and Pulmonary Section Medical Service, Veterans Affairs Ann Arbor Health System, Ann Arbor, MI, United States; 2 Division of Pulmonary and Critical Care Medicine, Department of Internal Medicine, University of Michigan, Ann Arbor, MI, United States; 3 Department of Surgery, University of Michigan, Ann Arbor, MI, United States

**Keywords:** bronchiolitis, deployment, fibrosis, lungs, MAPK, PD-L1, RSK1

## Abstract

**Background and aim:**

Deployment-related constrictive bronchiolitis (DRCB), a chronic fibrotic small airway disease, has been reported in military personnel following deployment to Southwest Asia and Afghanistan. Veterans diagnosed with DRCB indicate exposure to inhalational hazards, yet the molecular pathophysiology of this disorder remains enigmatic. Club cells are local progenitors critical for repair of small airway epithelium after inhalational injury. We have previously modeled DRCB using transgenic CC-DTA mice in which sustained club cell injury induces murine constrictive bronchiolitis (mCB) that recapitulates many of the histopathologic abnormalities observed in DRCB including peribronchiolar inflammation and fibrosis. The aim of the current study was to identify molecular pathways activated at the site of injury during the development of mCB.

**Methods:**

CC-DTA and control mice were exposed to doxycycline on protocol day 0–10 to induce club cell injury. Protein digital spatial profiling restricted to small airways was performed on lung sections harvested on protocol day 0, 10, and 20 and targeted a panel of 57 proteins focused on immune modulation and MAPK signaling.

**Results:**

Sustained club cell injury mediated small airway wall thickening due to enhanced deposition of subepithelial collagen fibers. Principal component analysis separated small airways of CC-DTA from control mice on day 10 and 20. Upregulated protein expression in small airways of CC-DTA versus control mice was found to be most numerous and prominent on day 20 and included an array of proteins involved in myeloid or lymphoid cell activation, signal transduction in response to extracellular stress stimuli, and regulation of cell proliferation, differentiation and survival. Immunofluorescence validated an increase in programmed death-ligand 1^+^ (PD-L1^+^) cells and in phosphorylated-p90 ribosomal S6 kinase^+^ (p-p90RSK^+^, a downstream effector of the MAPK/ERK pathway) epithelial cells in response to sustained club cell injury.

**Conclusion:**

We identified features of chronic innate and adaptive immune activation and aberrant collagen deposition within injured small airways during the development of mCB. Further, our findings implicate the PD-1/PD-L1 axis and the MAPK/ERK pathway in the pathogenesis of DRCB and may reveal potential therapeutic targets for combating this disease.

## Introduction

Deployment-related constrictive bronchiolitis (DRCB), a chronic, fibrotic small airway lung disease characterized by cough and exertional dyspnea leading to impaired exercise tolerance, has been identified in military personnel following deployment to Southwest Asia and Afghanistan (1–5). Development of DRCB has been associated with exposure to various airborne hazards including open-air military base burn pit emissions, sulfur dioxide (SO_2_), desert dust, and sandstorms (6). However, the cellular and molecular pathways triggered locally within small airways in response to these inhalational injuries, and the pathological mechanism through which they culminate in irreversible scarring are still unclear. Moreover, FDA-approved treatment for DRCB is currently unavailable and to date no clinical trials have been conducted. Lack of mechanistic understanding regarding the pathophysiology of this disease hinders our capacity to make much needed, clinically relevant progress toward combating DRCB.

To address these challenges, we have previously established two murine models of DRCB (7, 8). In an SO_2_ exposure model (7), murine constrictive bronchiolitis (mCB) was induced in C57BL/6 J mice by exposure to 50 ± 5 ppm SO_2_ for one hour/day for five consecutive days. In response to this inhalational injury, club cells (local progenitors lining the small airway epithelium) were triggered to proliferate and eventually differentiate into ciliated cells, while small airway wall thickness significantly increased due to subepithelial collagen deposition – a defining feature of DRCB. However, no peribronchiolar inflammation, a common attribute of DRCB, was detected using this exposure protocol.

In a sustained club cell injury model (8), mCB was induced in transgenic CC-DTA mice (9) by doxycycline administration for 10 consecutive days which induced targeted diphtheria toxin A (DTA) expression within airway club cells. We showed that sustained targeted club cell injury promoted acute accumulation of various myeloid cell subsets and increased local production of pro-inflammatory cytokines. Transition to a chronic phase was characterized by upregulated expression of oxidative stress-associated genes, activation of TGFβ, accumulation of alternatively activated macrophages, and development of peribronchiolar fibrosis. Interestingly, depletion of alveolar macrophages in doxycycline-exposed CC-DTA mice decreased TGFβ activation and ameliorated peribronchiolar fibrosis. Notably, sustained club cell injury mediated small airway epithelial metaplasia, wall thickening, peribronchiolar immune infiltrates, and respiratory bronchiolitis (clusters of intraluminal airway macrophages). We consider the sustained club cell injury model to be a useful framework to study DRCB as it represents a common pathway downstream of various inhalational hazards present in deployment zones and it recapitulates key abnormalities observed in DRCB. In the current study, we sought to identify key local cellular and molecular pathways that may promote peribronchiolar fibrosis in response to sustained club cell injury.

Constrictive bronchiolitis is a focal disease affecting the small airways of the lung. Hence, in whole lung-based studies the intensity of critical local signals may be dampened. To overcome this challenge, we employed GeoMx protein, small airway-restricted, digital spatial profiling (DSP, by Nanostring/Bruker). This recently developed platform allows comparative analysis of protein expression levels at spatially designated regions of interest (ROIs) using formalin-fixed paraffin-embedded tissue sections. To this end, a panel of proteins is targeted by a cocktail of antibodies attached to digital barcodes (DNA indexing oligos) via a UV photocleavable linker. ROI-restricted UV light exposure of the slides releases the barcodes to allow data acquisition. We selected to target 57 proteins involved in immune modulation and MAPK signaling. Immune-related proteins were chosen since previous studies by us (8) and others (10, 11) using murine models and clinical samples implicated immunopathology in the development of constrictive bronchiolitis. MAPK signal transduction proteins were examined as several reports in the scientific literature (12–14) highlighted the central role of these fundamental pathways in regulating and promoting fibrogenesis in the lung, heart, and kidney.

Findings from the current study demonstrate that ROI-restricted DSP is a powerful tool in identifying and monitoring focal alterations in protein expression levels during the development of mCB. Moreover, upregulated expression of two proteins detected by DSP was validated using immunofluorescence staining implicating programmed death-ligand 1 (PD-L1) and phosphorylated-p90 ribosomal S6 kinase (p-p90RSK) in promoting collagen fiber deposition within small airway walls in response to sustained club cell injury.

## Materials and methods

### Mice

Triple transgenic Scgb1a1rtTA/tetOCre/R26:lacZ/DT-A (CC-DTA) mice reported by Perl et al. (9) have a reverse tetracycline transactivator gene driven by the club cell secretory protein (CCSP) promoter, a Cre recombinase gene under the control of a tet operator sequence, and a lox-P activated DTA gene. Exposure of CC-DTA mice to doxycycline activates DTA expression only in club cells leading to targeted autonomous cell death. CC-DTA mice were bred on site and housed under specific pathogen-free conditions in the Animal Care Facility at the Ann Arbor Veterans Affairs Health System. Animals were provided with food and water ad libitum, routine husbandry, standard housing, and 12-h light/dark cycle. This study was carried out in accordance with the recommendations in the Guide for the Care and Use of Laboratory Animals of the National Institutes of Health. The animal protocol was approved by the Institutional Animal Care and Use Committee and by the Biosafety Committee at the Veterans Affairs Ann Arbor Health System, Ann Arbor, Michigan.

### Doxycycline exposure

Sustained club cell injury leading to mCB was induced by exposure of CC-DTA mice to doxycycline via their food (625 mg doxycycline/kg chow; Teklad, Envigo, Madison, WI) for 10 consecutive days, on protocol day zero till ten (8, 9). Single transgenic CRE littermate mice served as control groups. Mice were euthanized for lung harvest on protocol day 0 (no doxycycline exposure), 10, and 20. Euthanasia was performed by isoflurane overdose using a drop jar followed by bilateral pneumothorax. To this end, mice were placed in a closed jar containing a gauze pad soaked in 0.3–0.4 mL of isoflurane. Isoflurane was administered by vapor inhalation, and the gauze pad was placed in a tissue cassette to eliminate direct contact between the animal and the anesthetic fluid. Mice were monitored for breathing and removed from the jar 2 min after breathing had ceased. This procedure was performed in a fume hood.

### Morphometric analysis and immunofluorescence

The lungs of mice were inflation-fixed *in situ* via the trachea with 10% neutral buffered formalin (NBF; Thermo Fisher Scientific, Waltham, MA). Lung lobes were then harvested, further fixed in 10% NBF, processed and embedded in paraffin. Four μm sections were stained with picrosirius red (PSR) and for immunofluorescence according to standard laboratory procedures. The following primary antibodies were used: rabbit anti-PD-L1 (D5V3B, 1:100; Cell Signaling Technologies, Danvers, MA), rabbit anti-phospho-RSK1 (Thr359, Ser363, SU03-65, 1:100; Invitrogen, Carlsbad, CA), rat anti-CD45-AF647 (EM-05, 1:100; Novus Biologicals, Centennial, CO), mouse anti-pan cytokeratin (C-11, 1:100; Abcam, Waltham, MA), rabbit anti-club cell secretory protein-AF647 (CCSP or uteroglobin, 122, 1:100; Novus Biologicals), and mouse anti-acetylated tubulin (6-11B-1, 1:100; Sigma Aldrich, St. Louis, MO). Secondary antibodies included: goat anti-rabbit-AF Plus 488, goat anti-mouse-AF Plus 555, goat anti-rabbit-AF Plus 647, and goat anti-rat-AF 647 (all at 1:200, Invitrogen). Sections were quenched and mounted using TrueVIEW autofluorescence quenching kit with DAPI (Vector Laboratories, Newark, CA). Imaging was performed using a Keyence (Itasca, IL) BZ-X810 All-in-One fluorescence microscope.

Histological evaluation, morphometric measurements, collagen fiber analysis, and immunofluorescence quantification were performed on random lung sections (five lobes per lung section per mouse) of 2–4 mice per group. All lung lobes were scanned, and pictures of all or ten randomly selected small airways per lobe were photographed and analyzed. Small airways were defined as having an internal diameter of 45–400 μm.

To assess the extent of peribronchiolar fibrosis in the lungs of CC-DTA mice exposed to doxycycline, small airway wall thickness was quantified. To this end, the area (μm^2^) of subepithelial collagen deposition in PSR-stained lung sections was determined by demarcating the basement membrane and the outer edge of the airway adventitia. For each small airway analyzed, the area of collagen deposition was normalized to the length of the subepithelial basement membrane (μm). Morphometric analysis was performed using ImageJ2 software (National Institutes of Health, Bethesda, MD).

To quantify the number of collagen fibers in the wall of small airways, fluorescent microscope images of small airways from PSR-stained lung sections were analyzed using CurveAlign (7, 15). To measure the mean width and length of collagen fibers in the wall of small airways, fluorescent microscope images of small airways from PSR-stained lung sections were analyzed using CT-FIRE (7, 16).

In lung sections stained using immunofluorescence, the percentage of PD-L1^+^ or p-RSK1^+^ cells in or immediately surrounding small airways was calculated using the following formula: [(PD-L1^+^ or p-RSK1^+^DAPI^+^/DAPI^+^) * 100]. Additionally, the number of PD-L1^+^ or p-RSK1^+^DAPI^+^ cells was enumerated and normalized to the total area of the small airway (mm^2^). Cells were enumerated and the total area of the small airway was determined using ImageJ2 software.

### Protein digital spatial profiling (DSP)

Formalin-fixed paraffin-embedded lung sections of doxycycline-exposed CC-DTA and control mice harvested on protocol day 0 (no injury), 10 (acute injury phase), and 20 (chronic fibrotic phase) underwent GeoMx mouse protein DSP by NanoString (Seattle, WA) through their Technology Access Program using a panel of 57 antibodies targeting proteins focused on immune modulation (cell typing, profiling, and activation status) and MAPK signaling. Profiling of protein expression in these lung sections was restricted to certain small airways designated as ROIs. ROIs were selected based on custom immunofluorescence targeting pan cytokeratin (AE-1/AE-3, Novus Biologicals), CCSP (polyclonal, Abcam), CD45 (EM-05, Novus Biologicals), collagen VI (EPR17072, Abcam) and a nuclear marker. In lung sections of day 10 and 20 CC-DTA mice, selected ROIs displayed patchy CCSP lining of the small airway epithelium and leukocyte and/or fibrosis markers. Relative protein expression was quantified using an nCounter instrument. Data were analyzed using the GeoMx DSP Data Analysis software according to the manufacturer’s instructions and built-in statistical analyses. Data were normalized for barcode hybridization efficiency, ROI area, the geometric mean of isotype control antibodies, and expression of housekeeping genes. DSP was performed on seven small airways (ROIs) from each lung (five lobes/lung) and included two mice per group.

### Statistical analysis

Data in bar graphs are presented as mean ± standard error of the mean (SEM). Data were evaluated by unpaired, two-tailed t-test or two-way analysis of variance (ANOVA) followed by Tukey’s multiple comparisons test. Statistical analysis was performed using Graph-Pad Prism software version 10. *p* values < 0.05 were considered statistically significant. For DSP data, > 2.0- or < −2.0-fold changes in protein expression and -log10p value > 1.3 were considered statistically significant.

## Results

### Sustained club cell injury leads to the development of peribronchiolar fibrosis by augmenting collagen fiber deposition

**Figure 1:**
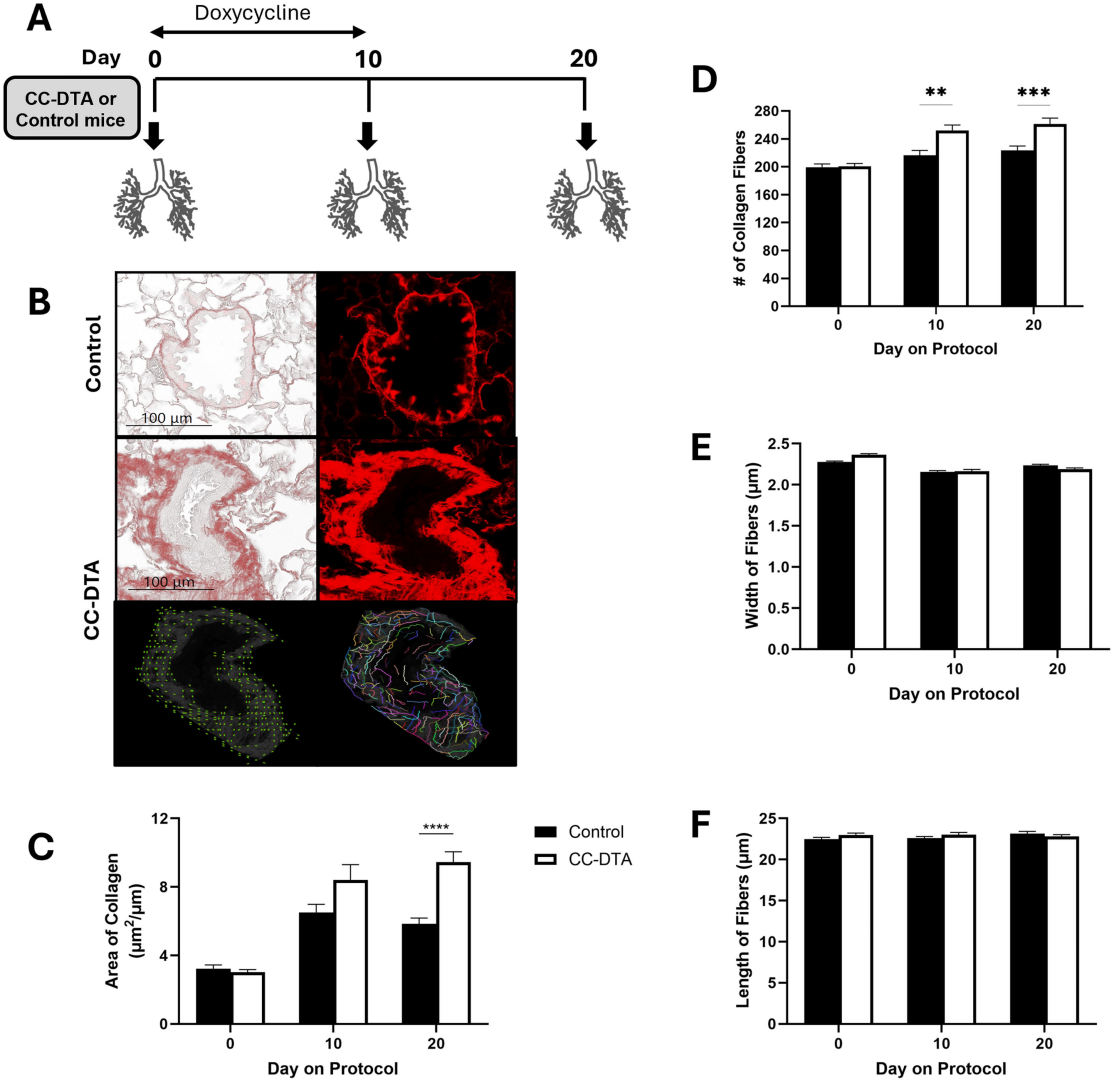
Sustained club cell injury induces peribronchiolar fibrosis through enhanced deposition of collagen fibers. **(A)** Schematic representation of study design. Cohorts of CC-DTA and control mice were exposed to doxycycline for ten consecutive days on protocol days zero to ten. Lungs were harvested on protocol day 0 (no doxycycline exposure), 10, and 20 for evaluation. (**B**, upper and middle panels) Representative phase-contrast (left panels) and fluorescent (right panels) microscopy images of small airways from PSR-stained lung sections of a control (upper panels) and CC-DTA (middle panels) mice exposed to doxycycline for ten consecutive days and harvested on protocol day 20. Note prominent red staining surrounding small airway of CC-DTA, but not control, mouse. (**B**, lower panels) The fluorescent microscopy image of a small airway from a CC-DTA mouse at day 20, shown in the right middle panel of **B**, was analyzed for number of collagen fibers using CurveAlign and for mean width and length of collagen fibers using CT-FIRE. CurveAlign-generated schematic representation of the number of collagen fibers identified in the image is shown in the left lower panel. CT-FIRE-generated schematic representation of the width and length of collagen fibers identified in the image is shown in the right lower panel. **(C)** Morphometric analysis of small airway wall thickness in lung sections from CC-DTA and control mice at the indicated protocol time points. The area, determined by demarcating the basement membrane and the outer edge of the airway adventitia, was normalized to the length of the subepithelial basement membrane. **(D)** The number of collagen fibers in the wall of small airways in PSR-stained lung sections of mice at the indicated protocol time points was quantified using CurveAlign. **(E,F)** The mean width **(E)** and length **(F)** of collagen fibers in the wall of small airways in PSR-stained lung sections of mice at the indicated protocol time points were quantified using CT-FIRE. **(C–F)** Data represent mean ± SEM of 57–107 small airways derived from two mice per group. **p < 0.01, ***p < 0.001, ****p < 0.0001; two-way ANOVA followed by Tukey’s multiple comparisons test.

Club cells serve as local progenitors within the epithelium of small airways in the lung. Under homeostatic conditions, they rarely proliferate. In response to acute injury, they possess a remarkable capacity for scarless regeneration. However, in response to repeated or sustained insult, this regeneration capacity may become exhausted, culminating in the development of peribronchiolar fibrosis, the defining feature of constrictive bronchiolitis. To study the molecular underpinning of this detrimental process, we employed the transgenic CC-DTA mouse model, in which DTA expression is induced by doxycycline and restricted to club cells (9). CC-DTA and control mice were exposed to doxycycline for ten consecutive days, and lungs were harvested from cohorts of mice on protocol day 0 (no injury), 10 and 20 (Figure 1A). As expected (8, 9), evaluation of PSR-stained lung sections of CC-DTA versus control mice at day 20 showed markedly enhanced subepithelial collagen deposition in small airway walls, i.e., constrictive bronchiolitis (Figure 1B, upper and middle panels). Morphometric analysis confirmed significant increase (> 60%) in the width of small airway walls in CC-DTA versus control mice at day 20 (Figure 1C). Prior work by us and others (8, 9) have shown that peribronchiolar fibrosis in this mouse model persists recapitulating the chronic histopathological findings in DRCB.

To further characterize collagen deposition in the wall of small airways in response to sustained club cell injury, the number of collagen fibers per small airway wall was quantified using CurveAlign analysis of fluorescent microscope images of small airways from PSR-stained lung sections (Figure 1B, left lower panel). Increased number of collagen fibers per small airway wall was detected in lungs of CC-DTA versus control mice at protocol day 10 and 20 (Figure 1D). The mean width and length of collagen fibers within the wall of small airways was determined using CT-FIRE analysis of fluorescent microscope images of small airways from PSR-stained lung sections (Figure 1B, right lower panel). No statistically significant differences in either mean width or length of collagen fibers within small airway walls were detected in lungs of CC-DTA versus control mice at the time points studied (Figures 1E,F). These results show that sustained club cell injury in mice promotes deposition of collagen fibers in small airway walls leading to increased small airway wall thickness. Interestingly, the number of collagen fibers, rather than their width or length, accounted for this finding.

### Sustained club cell injury alters local protein expression during the acute and chronic phase of mCB

**Figure 2:**
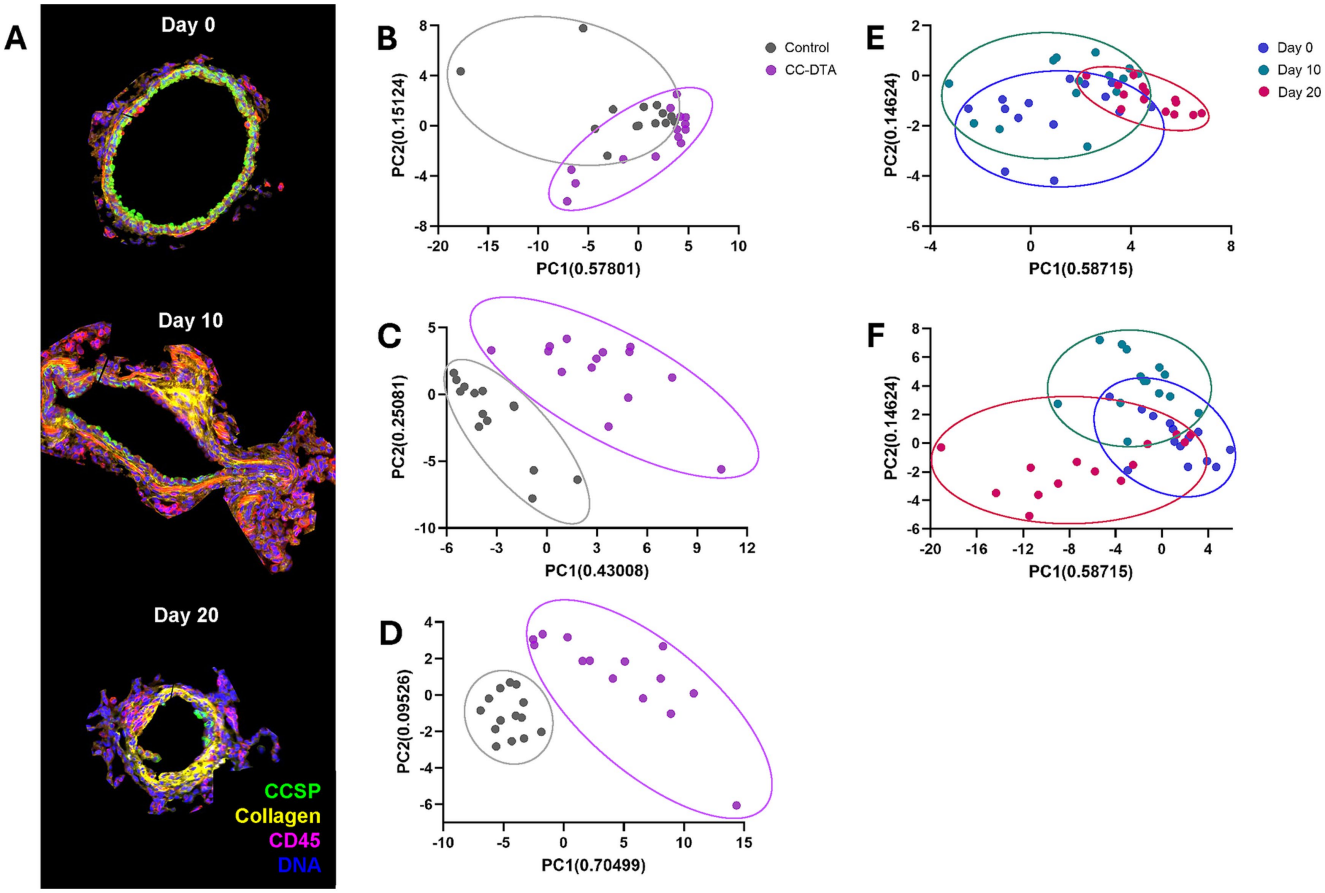
Sustained club cell injury alters local protein expression throughout the development of peribronchiolar fibrosis. Lung sections of doxycycline-exposed CC-DTA and control mice at protocol day 0, 10, and 20 underwent small airway-restricted DSP targeting 57 proteins. Immunofluorescence for CCSP, CD45, and collagen facilitated selection of seven small airways per lung as designated ROIs for DSP analysis. **(A)** Representative ROIs from a doxycycline-exposed CC-DTA mouse at the designated time points. Note intact club cell lining of small airway at day 0; partial club cell depletion, immune infiltrates, and subepithelial collagen deposition at day 10 and 20. **(B–D)** Principal component analysis (PCA) plots of small airways from CC-DTA and control mice on protocol day 0 **(B)**, 10 **(C)**, and 20 **(D)**. **(E)** PCA plots of small airways from control mice at protocol day 0, 10, and 20. **(F)** PCA plots of small airways from CC-DTA mice at protocol day 0, 10, and 20.

To identify central molecular pathways modulated locally in response to sustained club cell injury, we performed protein DSP on lung sections of CC-DTA and control mice obtained on protocol day 0 (no exposure to doxycycline - no club cell injury), 10 (after exposure to doxycycline for ten consecutive days - immediately after sustained club cell injury), and 20 (ten days after cessation of doxycycline exposure – chronic phase after sustained club cell injury). DSP targeted 57 proteins involved in immune modulation and MAPK signaling and was restricted to small airways designated as ROIs. ROIs were identified and selected via immunofluorescence for pan cytokeratin (epithelial cell marker, done on serial slides), CCSP (club cell marker), CD45 (leukocyte marker), collagen (surrogate marker for fibrosis), and a nuclear dye. Representative ROIs from a doxycycline-exposed CC-DTA mouse at the above-mentioned time points are shown in Figure 2A. On protocol day 0, club cells (CCSP^+^) lined the entire inner circumference of the small airway and minimal presence of leukocytes (CD45^+^) and collagen were observed. In contrast, on protocol day 10 and 20, partial club cell depletion, immune infiltrates, and subepithelial collagen deposition were evident.

**Figure 3:**
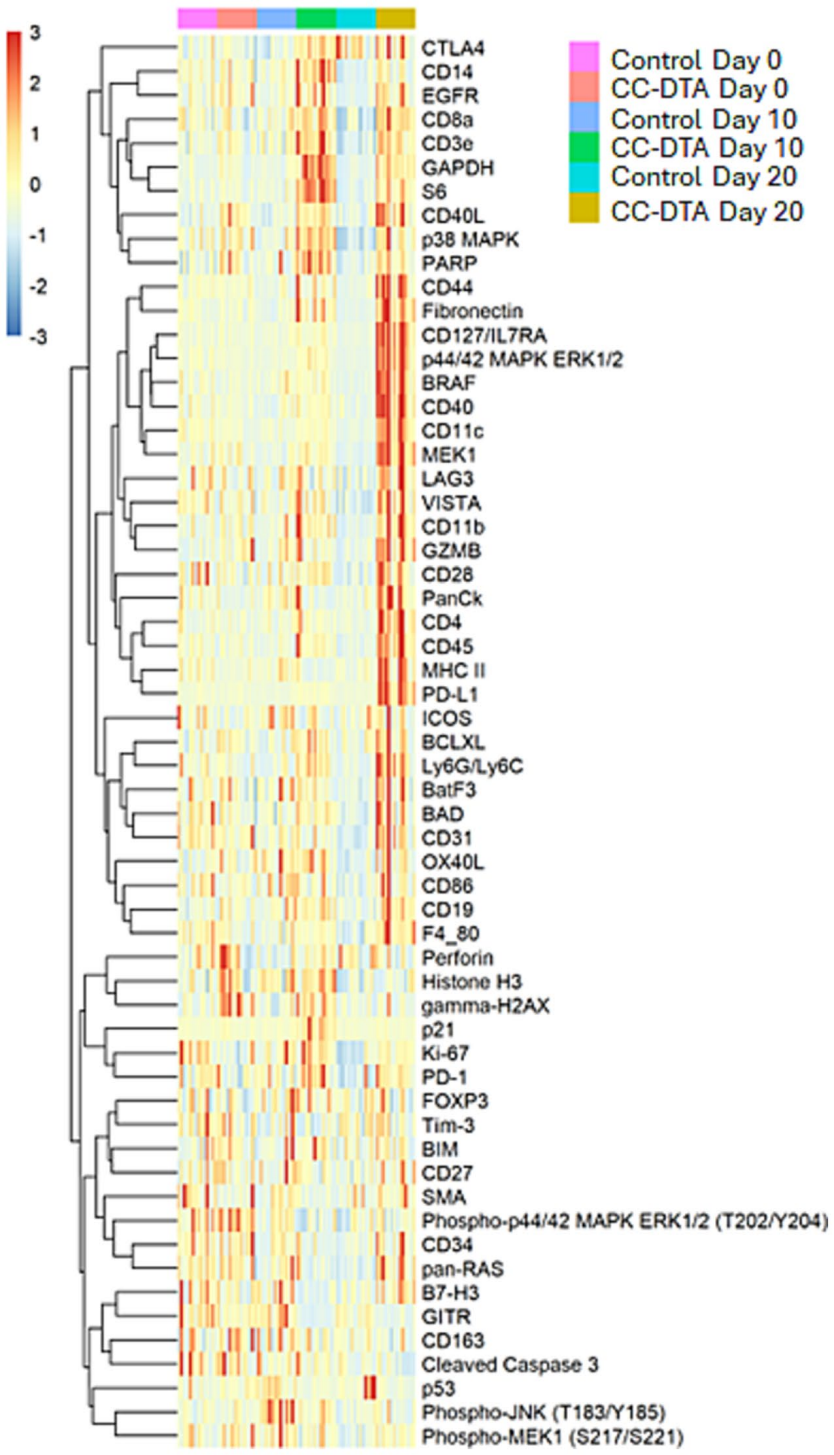
Local upregulated protein expression during the development of peribronchiolar fibrosis is most numerous and pronounced at the chronic fibrotic phase. Heat map depicting differential protein expression in small airways of CC-DTA versus control mice at protocol day 0, 10, and 20.

**Figure 4:**
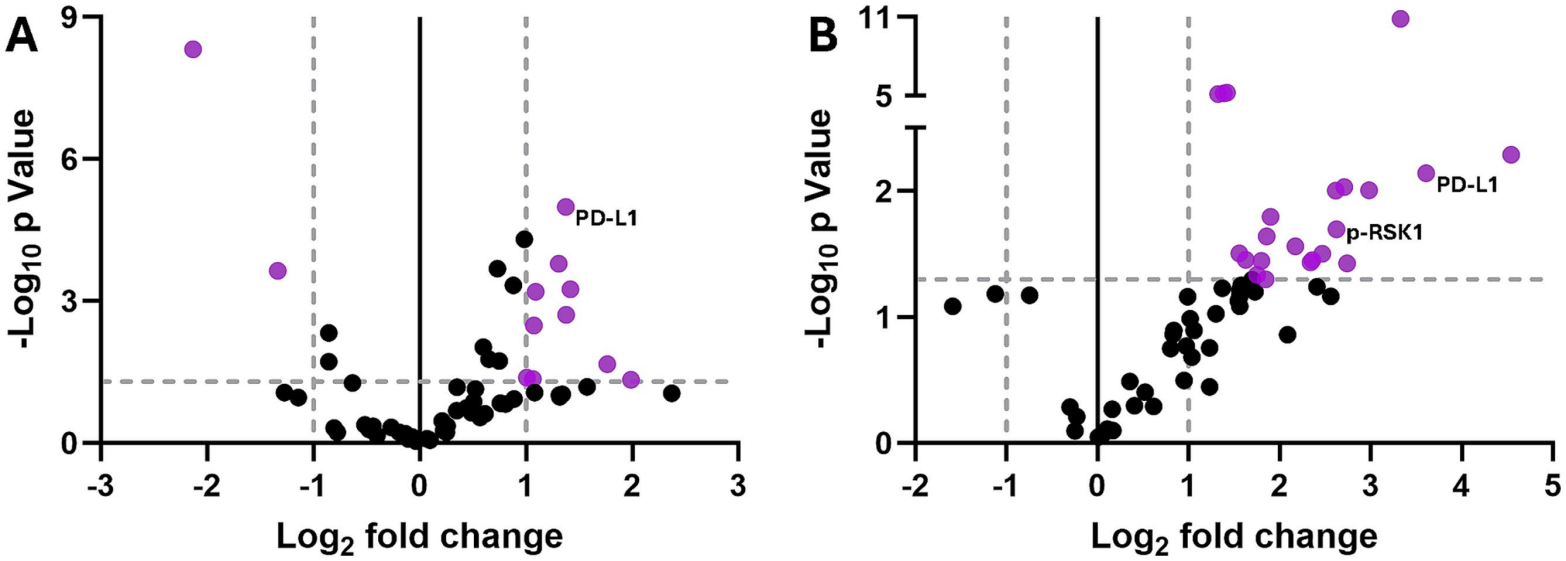
Peribronchiolar fibrosis is associated with local chronic differential protein expression. Volcano plots of protein DSP data showing differential expression in small airways of CC-DTA versus control mice at protocol day 10 **(A)** and 20 **(B)**. Vertical dotted lines depict log_2_ fold change in protein expression considered statistically significant. Horizontal dotted line depicts −log_10_ *p* value considered statistically significant. Gray dots represent proteins exhibiting no statistically significant differential expression relative to control airways. Purple dots represent proteins with statistically significant differential expression relative to control airways (at least twofold difference in expression relative to control airways and -log_10_ *p* value > 1.3).

Principal component analysis (PCA) plots of the protein DSP data showed that small airways of CC-DTA and control mice superimposed on day 0 (Figure 2B) but clearly separated on day 10 (Figure 2C), and 20 (Figure 2D). PCA plots also demonstrated that small airways of control mice from day 0, 10, and 20 superimposed (Figure 2E), whereas small airways of CC-DTA mice from day 0, 10, and 20 partially separated (Figure 2F). These findings indicate that in response to sustained club cell injury and throughout the development of peribronchiolar fibrosis, local protein expression levels assessed by this DSP panel were substantially modulated. To highlight this point, Figure 3 shows a heat map of differential protein expression within the panel used for DSP analysis and across the six experimental groups. Differences in protein expression between small airways of CC-DTA versus control mice on protocol day 0 appeared to be few and subtle but became more widespread and pronounced on day 10 and ultimately peaked at day 20. Moreover, most proteins in the DSP panel that were found to be modulated were upregulated. To better visualize these findings, volcano plots depicting the protein DSP data on protocol day 10 and 20 are shown in Figures 4A,B, respectively. Two proteins on day 10 and none of the proteins on day 20 were downregulated. In contrast, eight proteins on day 10 and twenty proteins on day 20 were upregulated. Interestingly, six proteins were identified as upregulated on both day 10 and day 20, and their fold change in expression further increased from day 10 to day 20. A complete list of proteins modulated on day 10 or/and day 20 is provided in Table 1. Proteins identified as upregulated in this DSP study are known to be involved in myeloid and/or lymphoid cell activation, cellular signal transduction in response to extracellular stress stimuli, and regulation of cell proliferation, differentiation and survival.

**Table 1:** Modulation of local protein expression during the development of peribronchiolar fibrosis.

Time of analysis	Protein	Fold change*	−log_10_ *p* value**
Day 10	p53	−2.523	3.644
	GITR	−4.382	8.323
	gamma-H2AX	2.675	3.253
	Fibronectin	2.477	3.793
	PD-L1	2.591	4.989
	CD44	2.597	2.708
	CD40	2.105	2.489
	CD14	2.091	1.355
	MEK1	2.133	3.201
	p38 MAPK	2.010	1.386
Day 20	Fibronectin	5.121	1.452
	PD-L1	12.169	2.141
	PD-1	2.939	1.504
	CD11c	6.526	2.030
	CD44	6.681	1.425
	CD127/IL7RA	6.127	2.004
	CD40	5.530	1.502
	Ly6G/Ly6C	4.503	1.561
	CD11b	3.730	1.795
	CD28	3.618	1.640
	CD8a	3.472	1.446
	CD3e	3.089	1.455
	CD19	2.615	5.210
	F4/80	2.677	5.257
	p44/42 MAPK ERK1/2	23.251	2.286
	MEK1	10.030	10.886
	BRAF	7.895	2.006
	Phospho-p90RSK (T359/S363)	6.156	1.697
	p38 MAPK	3.362	1.336
	PanCk	2.499	5.146

^*^A fold change >2 or <−2 was considered significant.

^**^A −log10 *p* value >1.3 was considered statistically significant.

### mCB is associated with an increase in PD-L1^+^ cells surrounding fibrotic small airways

**Figure 5:**
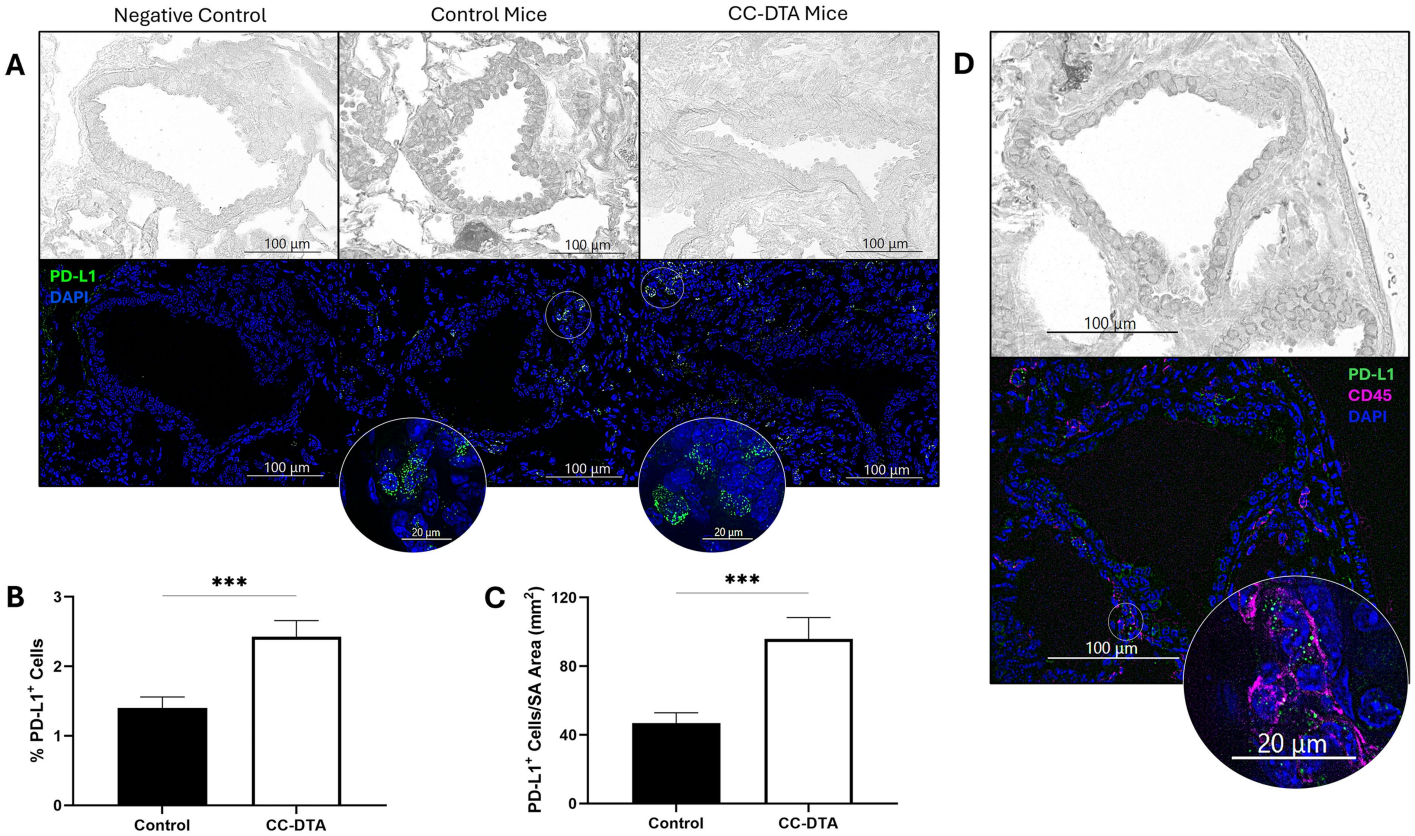
Peribronchiolar fibrosis is associated with an increase in PD-L1^+^ cells. **(A)** Representative phase-contrast (upper panels) and fluorescent (lower panels) microscopy images of small airways from lung sections of control (middle panels) and CC-DTA (right panels) mice exposed to doxycycline for ten consecutive days and harvested on protocol day 20. Lung sections were stained using an immunofluorescent antibody targeting PD-L1 and a nuclear dye (DAPI). Left panels were stained with secondary antibody only. Insets show magnification of areas designated by white circles. **(B)** The percentage of PD-L1^+^ cells in each small airway was determined using the following equation: (PD-L1^+^DAPI^+^/DAPI^+^) * 100. **(C)** The number of PD-L1^+^DAPI^+^ cells was enumerated and normalized to the total area of the small airway (SA, mm^2^). (B and C) Data represent cumulative results of two independent experiments consisting of 136–168 small airways derived from 4 mice per group. *** *p* < 0.001, unpaired, two-tailed *t*-test. **(D)** Lung sections were stained using immunofluorescent antibodies targeting PD-L1, CD45, and DAPI. Note PD-L1^+^CD45^+^ and PD-L1^+^CD45^−^ cells.

Intriguingly, the results of our small airway-restricted DSP for immune-related proteins (see Table 1) showed a consistent (both day 10 and day 20) and pronounced (>12-fold on day 20) increase in PD-L1 expression in CC-DTA versus control mice. To validate these findings, we performed immunofluorescence targeting PD-L1 on day 20 lung sections of doxycycline-exposed CC-DTA and control mice derived from two additional independent experiments. As shown in Figure 5A, few PD-L1^+^ cells were observed in small airways of control mice but positive cells appeared more prevalent in CC-DTA mice. Quantification of the percentage of PD-L1^+^ cells in small airways showed a significant increase in CC-DTA versus control mice (Figure 5B). Normalization of the number of PD-L1^+^ cells to the small airway area yielded similar results (>2-fold increase, Figure 5C). Multiplex immunofluorescence for CD45 allowed us to identify some of the PD-L1^+^ cells as leukocytes, but many PD-L1^+^CD45^−^ cells were observed (Figure 5D). These findings support and complement the DSP results as they demonstrated that enhanced PD-L1 protein expression in small airways of CC-DTA versus control mice at the chronic phase of fibrosis (protocol day 20) can be accounted for by accumulation of PD-L1^+^ cells.

### mCB is associated with enhanced expression of p-p90RSK by airway club and ciliated cells

**Figure 6:**
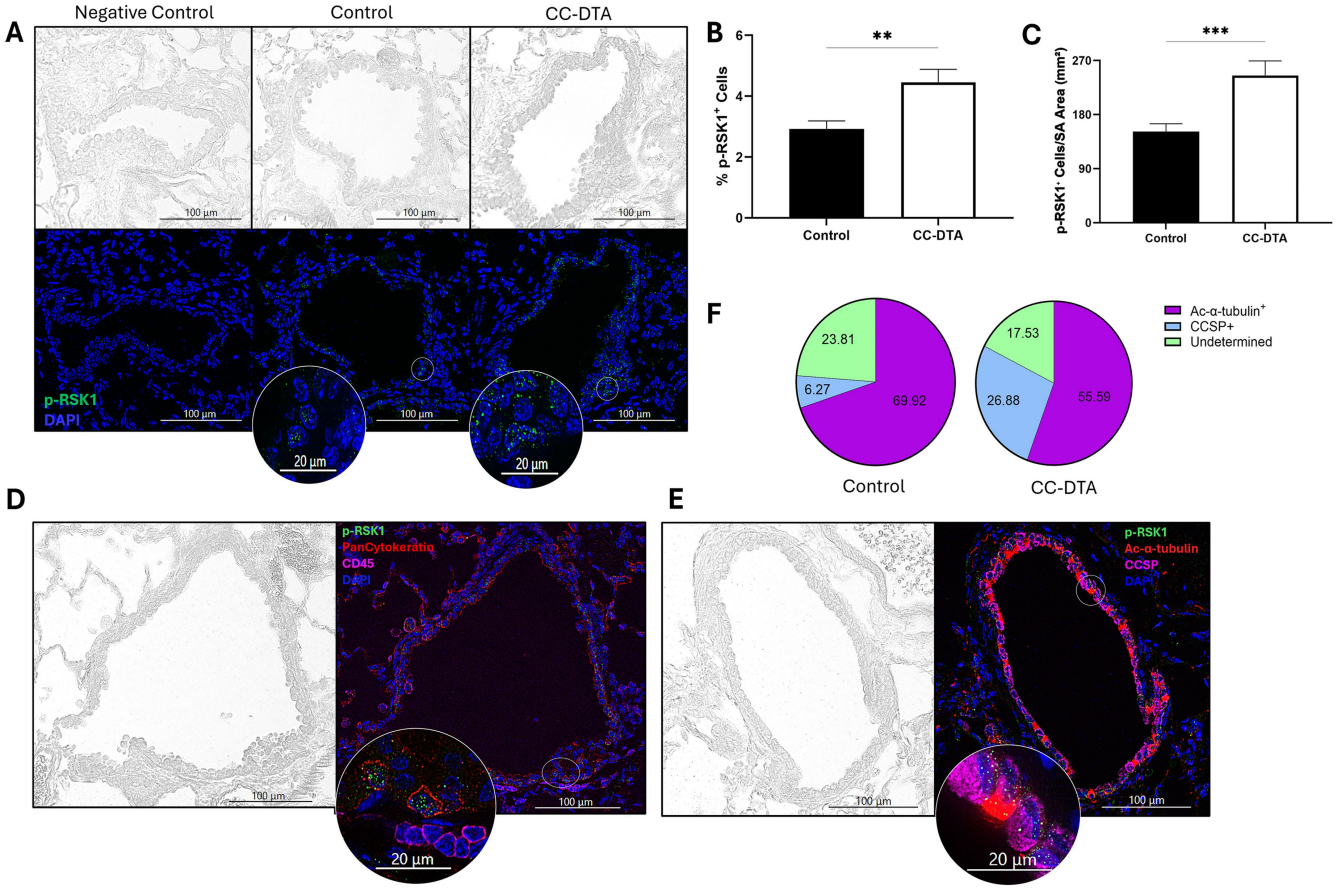
Peribronchiolar fibrosis is associated with enhanced p-RSK1 expression in epithelial cells lining the small airways. **(A)** Representative phase-contrast (upper panels) and fluorescent (lower panels) microscopy images of small airways from lung sections of control (middle panels) and CC-DTA (right panels) mice exposed to doxycycline for ten consecutive days and harvested on protocol day 20. Lung sections were stained using an immunofluorescent antibody targeting p-RSK1 and a nuclear dye (DAPI). Left panels were stained with secondary antibody only. Insets show magnification of areas designated by white circles. **(B)** The percentage of p-RSK1^+^ cells in each small airway was determined using the following equation: (pRSK1^+^DAPI^+^/DAPI^+^) * 100. **(C)** The number of p-RSK1^+^DAPI^+^ cells was enumerated and normalized to the total area of the small airway (SA, mm^2^). **(C,D)** Data represent cumulative results of two independent experiments consisting of 110–147 small airways derived from 4 mice per group. **** *p* < 0.0001, unpaired, two-tailed *t*-test. **(D,E)** Lung sections were stained using immunofluorescent antibodies targeting p-RSK1, pan cytokeratin **(D)**, CD45 **(D)**, acetylated-alpha-tubulin (ac-*α*-tubulin, **E**), CCSP **(E)**, and DAPI. The insets show a magnification of the areas designated by white circles. Note p-RSK1^+^pan cytokeratin^+^ **(D)**, p-RSK1^+^CCSP^+^ **(E)**, and p-RSK1^+^ acetylated-alpha-tubulin^+^ **(E)** cells. **(F)** Pie charts depicting the percentage of p-RSK1^+^ac-α-tubulin^+^ and p-RSK1^+^ CCSP^+^ in doxycycline-exposed CC-DTA and control mice on protocol day 20.

Results of our small airway-restricted DSP for MAPK signaling proteins (see Table 1) showed substantially enhanced expression of BRAF (>7.8-fold), MEK1 (>10-fold), and p44/42 MAPK ERK1/2 (>23-fold) in CC-DTA versus control mice on day 20. However, levels of phosphorylated-MEK1 (S217/S221) and phosphorylated-p44/42 MAPK ERK1/2 (T202/Y204) were not elevated (data not shown). Nevertheless, p-p90RSK (T359/S363), a major direct downstream effector of the MAPK/ERK pathway, was upregulated more than 6-fold. To confirm this latter finding, we conducted immunofluorescence targeting p-RSK1 on lung sections obtained at protocol day 20 from doxycycline-exposed CC-DTA and control mice derived from two additional independent experiments. Few p-RSK1^+^ cells were detected in small airways of control mice but seemed more prevalent in CC-DTA mice (Figure 6A). We then validated that the percentage of p-RSK1^+^ cells in small airways of CC-DTA versus control mice was significantly increased (Figure 6B) and further demonstrated that the number of p-RSK1^+^ cells normalized to the small airway area showed similar results (>1.6-fold increase, Figure 6C). We noted that p-RSK1^+^ cells localized to the epithelial basement membrane and multiplex immunofluorescence identified most (~98%) p-RSK1^+^ cells as epithelial (pan cytokeratin+) cells, whereas none were leukocytes (CD45^+^, Figure 6D). Further immunofluorescence studies revealed that both p-RSK1^+^CCSP^+^ and p-RSK1^+^alpha-acetylated-tubulin^+^ cells were present in the epithelium of small airways indicating expression by club as well as ciliated cells (Figure 6E). Interestingly, the percentage of p-RSK1^+^CCSP^+^ cells was increased in CC-DTA versus control mice (*p* < 0.0001, Figure 6F). Collectively, these findings identify local activation of the MAPK/ERK/p90RSK pathway in response to club cell injury, which may promote fibrogenesis and the development of mCB.

## Discussion

We have previously shown that exposure of CC-DTA mice to doxycycline for ten consecutive days mediates sustained club cell injury resulting in subepithelial collagen deposition culminating in increased small airway wall thickness - the defining histopathologic feature of DRCB (8, 9). In the current study, we used this murine model of DRCB to elucidate cellular and molecular pathways activated locally at the site of small airway injury. Results demonstrate that peribronchiolar fibrosis in mCB stems from increased deposition of subepithelial collagen fibers. Development of mCB was associated with upregulation of proteins involved in myeloid or lymphoid cell activation, signal transduction in response to extracellular stress stimuli, and regulation of cell proliferation, differentiation and survival. We further identified upregulation of PD-L1 amongst peribronchiolar cells and increased expression of p-p90RSK by airway club and ciliated cells indicating that immunomodulatory and the MAPK pathways may contribute to mCB pathogenesis and thus constitute potential therapeutic targets to be explored in future studies.

The results of this study shed new light on the pathogenesis of mCB at the cellular and molecular level. First, we validated and extended our original findings by demonstrating that subepithelial fibrosis results from an increase in the number, but not width or length, of collagen fibers deposited in small airway walls. In contrast, using a different murine model of DRCB, we found that SO_2_ exposure of mice induced peribronchiolar fibrosis by increasing the width, length, and number of collagen fibers in small airway walls (7). Further studies aimed at gaining better insight into molecular processes regulating collagen fiber deposition, particularly within small airway walls, may prove informative for DRCB as well as other fibrotic small airway diseases.

To focus our analysis on the microanatomic site of club cell injury, we used ROI-restricted DSP to compare local levels of protein expression at small airways during the development of peribronchiolar fibrosis. Immediately after sustained club cell injury (protocol day 10), but not before injury (day 0) or ten days after cessation of injury (day 20), we detected an increase in gamma-H2AX, the phosphorylated form of the histone protein H2AX. Gamma-H2AX is an early cellular response to the induction of DNA double-strand breaks and thus serves as a highly specific and sensitive molecular marker for monitoring DNA damage and cellular injury (17). This finding aligns well with the kinetics of club cell injury induction in the CC-DTA mouse model and thus supports the validity of the DSP results.

We identified upregulated level of expression of fibronectin in small airways of CC-DTA versus control mice on both protocol day 10 and 20 (but not on day 0). Fibronectin, a high-molecular weight glycoprotein, is an early, important component of the extracellular matrix viewed as a marker of fibrosis. Furthermore, a certain form of cellular fibronectin, extra type III domain A, has been implicated in the development of pulmonary fibrosis since it regulates extracellular matrix deposition by serving as a scaffold that facilitates assembly of collagen and other matrix proteins, promotes differentiation of fibroblasts into myofibroblasts, and mediates TGF-*β* activation (18). Notably, as peribronchiolar fibrosis progressed, the fold change in fibronectin expression increased from 2.477 on day 10 to 5.121 on day 20. Once again, this result is in accordance with the established timeline of small airway scarring in the CC-DTA model and thus helps validate the DSP data set.

Our analysis of immune-associated proteins identified moderately (2.6–6.5-fold) enhanced local expression of immune phenotyping markers in small airways undergoing scarring (day 20). Upregulated expression levels of CD11c, CD11b, F4/80, and Ly6G/Ly6C likely imply macrophage-enriched immune infiltrates corroborating our prior report (8). Moreover, DSP revealed that these peribronchiolar immune infiltrates are also comprised of CD4^+^ and CD8^+^ T lymphocytes as well as B lymphocytes. Furthermore, increased levels of CD28, CD40, and CD44 strongly suggest immune activation. These findings are consistent with the chronic peribronchiolar inflammation described in the lungs of military personnel with DRCB (3). Specifically, Gutor et al. (2, 10) reported immune infiltrates containing activated CD4^+^ and CD8^+^ T lymphocytes within small airway walls. In a previous study (8), using multiparameter flow cytometry analysis and gene expression profiling, we detected accumulation of alternatively activated macrophages in lungs of CC-DTA mice during the chronic fibrotic phase (protocol day 20). Importantly, macrophage depletion led to reduced activation of TGF-*β* and ameliorated collagen deposition in small airway walls. Altogether, these findings indicate that sustained club cell injury leads to persistent peribronchiolar infiltrates of activated innate and adaptive immune cells.

Amongst the immune-related proteins interrogated in our comparative DSP study, the most striking upregulation was observed in expression of PD-L1. While no modulation of expression level was noted on protocol day 0, a higher than 2.5-fold and 12-fold increase were detected on day 10 and 20, respectively. PD-L1 [reviewed in (19)] functions as an immune checkpoint protein by suppressing adaptive immune responses to curb overreactions and induce tolerance critical for preventing autoimmunity. It binds to programmed cell death protein 1 (PD-1), also found to be upregulated on day 20 by almost 3-fold, transmitting an inhibitory signal that reduces both antigen-specific T cells proliferation and regulatory T cells apoptosis. PD-L1 is typically expressed in myeloid, lymphoid, and epithelial cells, but aberrant expression by other cells, particularly cancer cells, is a well-known mechanism by which tumors evade immune detection and eradication. Moreover, immune checkpoint inhibitors like anti-PD-1 and anti-PD-L1 antibodies have revolutionized immunotherapy of cancer in the past decade. The role of the PD-1/PD-L1 axis in fibrosis is less defined (20, 21). Nevertheless, in recent years several preclinical studies have shown that this axis promotes the development of pulmonary fibrosis and that blockade of the PD-1/PD-L1 pathway alleviates the severity of disease (22–26). While the underlying mechanisms remain uncertain, evidence suggest that aberrant expression of PD-L1 on human and mouse lung fibroblasts may inhibit autophagy and/or mediate effects through p53, FAK, Smad3, and/or *β*-catenin signaling pathways leading to myofibroblast transition (22–26). These reports implicate the PD-1/PD-L1 pathway in alveolar fibrosis, whereas our findings suggest this pathway may contribute to fibrosis within small airways. In light of these reports, future studies to further identify PD-L1^+^ cells and determine their role in the development of peribronchiolar fibrosis using the CC-DTA mouse model are warranted.

Small airway-restricted DSP of MAPK signaling proteins during the chronic fibrotic phase (protocol day 20) demonstrated that protein kinases of the MAPK/ERK1/2 pathway were highly upregulated. While increased expression of pan-RAS did not reach statistical significance, levels of BRAF, MEK1, and p44/42 MAPK ERK1/2 were elevated more than 7.8-, 10-, and 23-fold, respectively. However, phosphorylated-MEK1 and phosphorylated-p44/42 MAPK ERK1/2 were not upregulated. Nevertheless, p-p90RSK, a major direct downstream effector of ERK, was upregulated more than 6-fold. p90RSK, a family of four isoforms of serine/threonine kinases, are known to regulate key cellular functions such as cell growth, proliferation, survival, migration, and invasion (27, 28). Dysregulated and hyperactivated p90RSK function has been associated with several human cancers, and therefore it has become a potential therapeutic target with small molecule kinase inhibitors currently being assessed in clinical trials (29, 30). Intriguingly, in pre-clinical studies, p90RSK has been reported to promote kidney (31), liver (32), and lung fibrosis (33). Moreover, in a bleomycin-induced lung fibrosis mouse model, inhibition of p90RSK was shown to ameliorate TGF-β1 signaling and the development of fibrosis within the lung interstitium (33). Our finding of increased p-p90RSK expression during small airway fibrogenesis supports further exploration of this signaling pathway in the pathogenesis of constrictive bronchiolitis.

The use of immunofluorescence to validate our DSP findings yielded several interesting findings. First, we verified an increased percentage of PD-L1^+^ and p-RSK1^+^ cells in small airways of CC-DTA mice that developed mCB (versus control mice). Second, this approach also visualized the location of positive cells within the small airway wall and facilitated their identification. We were able to identify some of the PD-L1^+^ cells in the small airways as CD45^+^ leukocytes, but many were CD45^−^ and require further characterization. In contrast, more than 98% of p-RSK1^+^ cells were epithelial cells, either club or ciliated cells, and none were leukocytes. Interestingly, in CC-DTA mice with mCB, a higher percentage of p-RSK1^+^ cells were club cells suggesting that injury may induce expression of p-RSK1 amongst club cells repopulating the injured epithelium.

Our findings may have additional relevance to ongoing efforts seeking to understand how sustained club cell injury results in regional fibrosis. In the CC-DTA transgenic model, club cell depletion and recovery is patchy, and it is intriguing to speculate on whether (and how) focal “patchiness” within the injured airway might directly or indirectly influence surrounding mesenchymal cell phenotypes. In addition, the degree to which CCSP, produced mainly by club cells, promotes or protects against fibrosis in this model remains uncertain. CCSP has recently been reported to dampen acute and chronic lung inflammation by various mechanisms (34). Interestingly, in obstructive lung diseases like asthma and COPD, CCSP levels tend to be reduced, whereas in restrictive lung diseases like idiopathic pulmonary fibrosis. Increased CCSP levels have been detected, perhaps due to club cell dysregulation (35). Yet this data is generally obtained in a static fashion once the disease phenotype has been established. Data from our initial study highlights dynamic temporal and spatial relationships between mRNA and protein levels of CCSP in both bronchoalveolar lavage fluid and serum. Collectively, our findings in the context of the published literature suggest that the contribution of club cell numbers, location, and CCSP levels to the development of region lung fibrosis warrants additional investigation. Validating our protein DSP findings using human lung sections derived from DRCB patients would be informative, yet the ability to conduct these experiments is limited due to a paucity of human tissue samples available for analysis.

The findings of our current study may prove relevant to other pre-clinical models of deployment-related respiratory disease. We and others have shown that sustained/repetitive SO_2_ exposure induces constrictive bronchiolitis, and our findings implicate club cell proliferation and differentiation in this process (7, 11). Multiple investigators have modeled additional putative deployment-relevant exposures including particulate matter, desert dust, metals/metalloids, gases, and simulated burn pit smoke. Trembley et al. (36) used whole-body inhalation of nano-sized carbon black (a surrogate for particulate matter) as a rat model of military burn pit exposure. Proinflammatory biomarkers were detected in various tissues including the lung, brain, arteries, and serum 24 h post exposure supporting induction of a murine chronic multi-symptom illness. Whole-body inhalation of carbon black and naphthalene was also recently used as a rat model of military burn pit exposures, and RNA sequencing performed 24 h post exposure identified modulation of inflammatory and metabolic gene expression in both lung and brain tissues (37). In the lungs, pathways related to proliferation and inflammation were found to be activated. The strengths of these approaches to modeling DRCB lie in a clinically relevant delivery system and the ability to simultaneously examine effects on multiple tissues. Conducting analysis at a later time point after exposure may reveal transition from inflammation to fibrosis. Intriguingly, intratracheal instillation of Iraqi dust in mice was shown after four weeks to induce lung inflammation with fibrosis via IL-2 upregulation and depletion of regulatory T cells (38).

Modeling DRCB by induction of sustained club cell depletion circumvents the need for exposures while exhausting the regenerative capacity of local progenitor cells lining the small airways leading to histological features consistent with DRCB (8). The acute inflammatory phase and chronic fibrotic phase observed in the CC-DTA transgenic model align with findings described in direct exposure models. Thus, it may prove informative to interrogate the PD-1/PD-L1 axis and the MAPK/ERK1/2/p90RSK signaling cascade in other exposure models to assess whether these pathways are shared amongst exposures that alter immune and fibrotic phenotypes. Our findings also suggest that the sustained club cell injury model might be well-suited to future studies incorporating other exposures to mimic “two-hit” insults that might occur in deployment zones.

To summarize, in this study, we detected, monitored, and quantified differential protein expressions in small airways of the lung during the development of peribronchiolar fibrosis in a murine model of DRCB. Our findings support an emerging paradigm that DRCB persists due to both chronic innate and adaptive immune activation promoting aberrant collagen deposition within injured small airways. Results further implicate sustained club cell injury in the activation of two distinct molecular pathways, the PD-1/PD-L1 axis and the MAPK/ERK1/2/p90RSK signaling cascade. Future pre-clinical studies are warranted to assess the therapeutic efficacy of blocking these pathways as this carries potential to inform the development of novel treatments for DRCB and other fibrotic small airway diseases.

## Data Availability

The raw data supporting the conclusions of this article will be made available by the authors, without undue reservation.
